# Magnetoencephalography reveals increased slow-to-fast alpha power ratios in patients with chronic pain

**DOI:** 10.1097/PR9.0000000000000928

**Published:** 2021-06-03

**Authors:** Bart Witjes, Sylvain Baillet, Mathieu Roy, Robert Oostenveld, Frank J.P.M. Huygen, Cecile C. de Vos

**Affiliations:** a Center for Pain Medicine, Department of Anesthesiology, Erasmus University Medical Center, Rotterdam, the Netherlands; b McConnell Brain imaging Centre, Montreal Neurological Institute, McGill University, Montreal, Canada; c Department of Psychology, McGill University, Montreal, Canada; d Donders Institute for Brain, Cognition and Behaviour, Radboud University, Nijmegen, the Netherlands; e NatMEG, Karolinska Institutet, Stockholm, Sweden

**Keywords:** Magnetoencephalography, Chronic pain, Biomarker, Functional brain imaging

## Abstract

**Introduction:**

Objective disease markers are a key for diagnosis and personalized interventions. In chronic pain, such markers are still not available, and therapy relies on individual patients' reports. However, several pain studies have reported group-based differences in functional magnetic resonance imaging, electroencephalography, and magnetoencephalography (MEG).

**Objectives:**

We aimed to explore spectral differences in resting-state MEG brain signals between patients with chronic pain and pain-free controls and to characterize the cortical and subcortical regions involved.

**Methods:**

We estimated power spectral density over 5 minutes of resting-state MEG recordings in patients with chronic pain and controls and derived 7 spectral features at the sensor and source levels: alpha peak frequency, alpha power ratio (power 7–9 Hz divided by power 9–11 Hz), and average power in theta, alpha, beta, low-gamma, and high-gamma bands. We performed nonparametric permutation *t* tests (false discovery rate corrected) to assess between-group differences in these 7 spectral features.

**Results:**

Twenty-one patients with chronic pain and 25 controls were included. No significant group differences were found in alpha peak frequency or average power in any frequency band. The alpha power ratio was significantly higher (*P* < 0.05) in patients with chronic pain at both the sensor and brain source levels. The brain regions showing significantly higher ratios included the occipital, parietal, temporal and frontal lobe areas, insular and cingulate cortex, and right thalamus.

**Conclusion:**

The alpha power ratio is a simple, promising signal marker of chronic pain, affecting an expansive range of cortical and subcortical regions, including known pain-processing areas.

## Abstract 2

Supplemental Digital Content is Available in the Text.

We report an increased slow-to-fast alpha power ratio of neurophysiological activity in chronic pain from known pain-processing areas, among others.

## 1. Introduction

Chronic pain is an ongoing sensation that lasts or recurs for more than 3 months.^30^ It can persist even after a causing event (eg, injury or illness) is healed, with a considerable negative impact on functionality and quality of life. The objective assessment of chronic pain is an ongoing challenge to patients and their caregivers. Subjective self-reports remain the primary measures reported. These, however, do not point at the physiological cause of the pain sensation and do not provide mechanistic insight and opportunities to comprehend the biological causes of interindividual variability in response to treatment. There is therefore a considerable clinical demand for objective biomarkers of chronic pain, which would mitigate the limitations of self-reported measures and provide tools to better assess and predict personal treatment efficacy. We here use the term *biomarker* as a characteristic measure of a biological process in health or disease, with potential to assess the biological response to an exposure or intervention.^11^ Functional neuroimaging techniques, such as functional magnetic resonance imaging (fMRI), electroencephalography (EEG), and magnetoencephalography (MEG), can be used to that end.

Evidence emerging from fMRI data indicates that subjective pain reports are associated with the activation of multiple pathways and brain areas, including somatosensory, dorsolateral prefrontal and cingulate cortices, insula, and thalamus.^1,13^ More direct measurements of neurophysiological brain activity with EEG or MEG provide the millisecond resolution required to clarify the nature of the association between the ongoing, subjective experience of pain and the underlying polyrhythmic fluctuations of brain network activity.^2^ Previous EEG and MEG studies have revealed aberrant spectral profiles of brain activity in patients with chronic pain, such as increased activity in the theta (4–8 Hz) frequency range, depressed activity in the alpha band (8–12 Hz), or a lower dominant peak frequency in task-free brain activity.^5,8,15,22,23,35^ In general, all these studies report relatively more power in the lower frequencies.

One explanatory model of aberrant spectral profiles in neurology is the integrative framework of thalamocortical dysrhythmia (TCD). The model of TCD suggests that there is a common mechanism underlying the spectral abnormalities in the theta and alpha bands in specific neurological disorders, such as tinnitus, Parkinson disease, depression, and pain.^17,21–23^. Perhaps, the aberrant spectral profiles reported in chronic pain (increased theta power, decreased alpha power, or lower alpha peak frequency) could all be described to TCD; if the dominant peak frequency shifts towards lower frequencies, alpha band power would decrease, whereas theta-band power would increase. To combine these spectral profiles, Schulman et al.^23^ used a ratio of power in the band 7 to 9 Hz to power in the band 9 to 11 Hz. An increase of this ratio could be caused by increased theta power, decreased alpha power, or shift of the dominant peak frequency towards lower frequencies.

Vanneste et al. reported TCD as involving the subgenual anterior cingulate cortex and insula across all these patient populations, whereas they reported more specific manifestations of TCD in the auditory cortex in tinnitus, the motor cortex in Parkinson disease, and in the somatosensory cortex in pain.^33^ In chronic pain, aberrant spectral profiles have been reported in the cingulate, insular, somatosensory, prefrontal, parietal and occipital cortices,^5,8,15,24,34,35^ and subcortical regions, primarily the thalamus.^14,21,23^

With our exploratory study, we aimed to advance the characterization of spectral differences in resting-state neurophysiological brain activity between patients with chronic pain and pain-free control participants. We hypothesized to detect spectral changes related to TCD and performed exploratory broad range (4–90 Hz) frequency analyses. We aimed to provide insight beyond previous reports limited to regions of interest known to be involved in pain processes and therefore conducted a series of whole-brain analyses using MEG source imaging.

## 2. Material and methods

### 2.1. Participants

Twenty-one patients with chronic pain and 29 age-matched and sex-matched control participants without pain enrolled in this study. The inclusion criteria for patients with chronic pain were a diagnosis of failed back surgery syndrome (FBSS) with chronic pain in the back or legs and being older than 18 years. Exclusion criteria were severe pain in other body parts or any other form of serious decline of general health. Control participants did not experience chronic pain or other neurological syndromes. Other moderate medical conditions were not an exclusion criterion. Patients were recruited from pain clinics in the Netherlands (Medical Spectrum Twente [Enschede], Erasmus University Medical Center [Rotterdam], Sint Maartenskliniek [Nijmegen]) and in Canada (Montreal Neurological Institute and Hospital and Hôpital Maisonneuve-Rosemont [Montreal]). Control participants were recruited through public announcements. Ethics approval was obtained from the Institutional Review Boards of the Montreal Neurological Institute (Montreal, Canada) and the CMO region Arnhem-Nijmegen (the Netherlands); all participants gave written informed consent to participate in the study.

### 2.2. Data acquisition

Before the MEG session, the participants were asked to fill in standard questionnaires concerning pain (the Brief Pain Inventory), generic health status (EuroQol 5 dimensions 5 levels), and anxiety and depression (the Hospital Anxiety and Depression Scale [HADS]). Each participant was offered to complete the questionnaires in their preferred language, as validated versions in Dutch, English, or French were available. We report the EQ5D data using EQ5D index values, with the crosswalk value set from the relevant country (the Netherlands or Canada).

Five-minute resting-state MEG recordings were collected at either the Montreal Neurological Institute (McGill University, Montreal, Canada) or the Donders Institute for Brain, Cognition and Behavior (Nijmegen, the Netherlands). The MEG systems, acquisition software, and measurement setups were identical at both locations. Participants were seated upright under the 275-channel whole-head MEG system (CTF, Coquitlam, BC, Canada) inside a passive magnetically shielded room. Before entering the magnetically shielded room, the participants changed to scrubs and were instructed to remove metal materials to ensure optimal data quality. The data sampling rate was 2400 Hz (built-in antialiasing filter below a 600-Hz cutoff), and third-order gradient compensation was applied for MEG noise reduction purposes. Reference signals for eye and cardiac artifacts were captured from horizontal and vertical electrooculograms and electrocardiograms. The participant's head position in the MEG helmet was registered using 3 head coils attached to 3 anatomical landmarks: nasion and left or right preauricular points. A 3D digitizer system (Polhemus Fastrak, Colchester, Vermont) was used to digitize the participant's head shape, the respective locations of the head-positioning coils, and anatomical landmarks. We collected a 2-minute empty room recording to capture environmental noise before every individual session and to inform the MEG source modeling process.^3^ Participants were instructed to sit still with their eyes open, focusing on a fixation cross.

### 2.3. Data analysis

#### 2.3.1. Preprocessing

All data processing was performed with Brainstorm using MATLAB version R2020a (the MathWorks, Natick, MA).^27^ Brainstorm is an open-source application freely available under the GNU general public license (http://neuroimage.usc.edu/brainstorm). We used the recommended processing pipeline for MEG preprocessing in Brainstorm, following good practice guidelines^10,28^: Resting-state recordings were visually inspected and cleaned from artifacts. Sensors with a low signal-to-noise ratio were excluded from further analysis (varying between 0 and 7 per participant). Powerline artifacts were attenuated with a notch filter at the powerline's frequencies and harmonics (50, 100, and 150 Hz for the Netherlands and 60, 120, and 180 Hz for Canada). A bandpass filter was applied to remove low-frequency (<1 Hz) and high-frequency (>200 Hz) noise. Cardiac and eye-blinking artifacts were attenuated with specific signal-space projections.^29,32^ Segments with other types of visible artifacts were excluded from further analysis.^28^

#### 2.3.2. Power spectral density analysis: magnetoencephalography sensors

We estimated the absolute power spectral density (PSD) of preprocessed individual MEG recordings from every sensor, using the Welch method with a 4-second Hamming window and 50% overlap. We first computed the average PSD across all sensors to inspect global group-wise differences. We then extracted 7 spectral features from individual sensor-wise PSD estimates: the alpha peak frequency, the alpha power ratio, and the average power in the theta (4–7.5 Hz), alpha (8–12.5 Hz), beta (13–30 Hz), low-gamma (30.5–60 Hz) and high-gamma (60.5–90 Hz) bands. The dominant peak frequency was identified for each sensor and participant by determining the frequency bin with the maximum power within the slightly broader search range of 7 to 13 Hz. As the dominant peak frequency was expected to shift to lower frequencies in patients with chronic pain,^5,8,15,22,23,35^ we hypothesized that the power in the low alpha band (7–9 Hz) would be increased for the power in the middle alpha band (9–11 Hz) in the group with chronic pain. We therefore derived the alpha power ratio measure (described by Schulman et al.^23^) to capture this anticipated effect, as the ratio between low alpha band and middle alpha band power.

#### 2.3.3. Power spectral density analysis: magnetoencephalography source mapping

The ICBM152 T1-weighted magnetic resonance imaging template^9^ was affinely transformed by Brainstorm to fit the digitized head shape of each participant. We then defined a 3D source grid consisting of 13,479 evenly distributed elementary current dipole sources across the entire brain volume. The MEG forward head model was obtained from the overlapping-sphere method also available in Brainstorm.^12^ We then derived the weighted minimum norm estimate of MEG source amplitudes at each brain location and each time point, with Brainstorm's default parameter values. We used the automated anatomical labeling (AAL) atlas^31^ registered to the ICBM151 cortical template to allocate each of the 13,479 grid points to 99 brain regions and subsequently considered for further analyses the 84 AAL atlas brain regions that consisted of more than 20 actual source locations in the MEG model. For each AAL brain region, the source time series were averaged across all brain locations in that region. Subsequently, the alpha peak frequency, alpha power ratio, and average power in the theta, alpha, beta, low-gamma, and high-gamma bands were computed from the corresponding PSDs, for each participant and for each of the 84 brain regions.

### 2.4. Statistical analysis

We used nonparametric permutation (2-tailed) *t* tests with 10,000 permutations to assess between-group differences in alpha peak frequency, alpha power ratio, and the average power in each of the frequency bands of interest both at the MEG sensor and source levels. We applied a false discovery rate (FDR) correction across the number of signals (either the number of sensors or the number of sources) and the number of frequency bands of interest.^4^ The observed effects were considered significant when the associated corrected *P* value was smaller than 0.05.

## 3. Results

### 3.1. Patient characteristics

One control participant withdrew from the study because the participant became anxious in anticipation to the MEG session. The data from 3 control participants were excluded because they reported severe muscle ache or a history of migraine or tinnitus only after completion of MEG recordings. In total, the data from 21 patients with FBSS (11 women, 48 ± 10 years) and 25 control participants (10 women, 49 ± 11 years) were included for further analysis.

Patient characteristics are shown in Table 1. For patients with pain, the average visual analogue scale (VAS) pain scores 24 hours before the MEG session were 6.1 ± 1.8 and the average VAS scores immediately before the MEG session were 5.0 ± 2.4. One participant from the control group did not report the EQ5D and the HADS, and one patient did not complete the Brief Pain Inventory questionnaire, therefore the rating of average pain experienced over the 24 hours before the MEG session was missing for this participant. The missing data were not imputed. The Hospital anxiety and depression scale scores were computed separately for the anxiety-related questions and for the depression-related questions. Medication usage was divided between opioids, neurotropic drugs (antiepileptic drugs, antidepressants, etc.), nonsteroidal anti-inflammatory drugs (NSAIDs), and other medications (not analgesic).

**Table 1:** Patient characteristics.

	Controls (n = 25)	Patients with pain (n = 21)	*P*
Age (y)	49 ± 11	48 ± 10	0.90
Sex (M/F)	15/10	10/11	0.26
Pain duration (y)	N/A	10 ± 9	—
Avg. pain (MEG) (VAS)	0 ± 0	5.0 ± 2.4	—
Avg. pain (24hours) (VAS)	0 ± 0	6.1 ± 1.8	—
EQ5D	959 ± 34	471 ± 271	<0.01
HADS-A	2 ± 2	8 ± 4	<0.01
HADS-D	1 ± 2	9 ± 4	<0.01
Medication			
Opiods (#)	0	13 (62%)	<0.01
Neurotropic drugs (#)	2 (8%)	15 (71%)	<0.01
NSAID (#)	1 (4%)	5 (24%)	<0.05

EQ5D, EuroQol 5 dimensions; HADS-A, Hospital Anxiety and Depression Scale–Anxiety; HADS-D, Hospital Anxiety and Depression Scale–Depression; MEG, magnetoencephalography; NSAID, nonsteroidal anti-inflammatory drug; VAS, visual analog scale.

Age, EQ5D, and HADS scores are presented as mean ± SD. Avg pain (MEG) is the VAS score right before the recording, avg. pain (24 hour) is the average VAS score in the 24 hours before the recording. We used an independent *t* test for differences in age, a Mann–Whitney *U* test for EQ5D and HADS scores, and a χ^2^ to test for sex.

### 3.2. Power spectral density analysis: magnetoencephalography sensors

**Figure 1.**
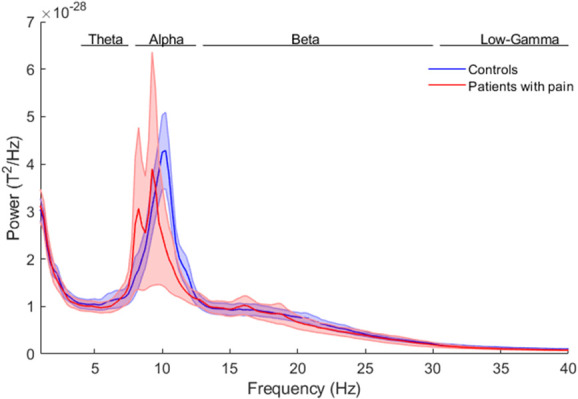
Whole-head average power spectral density estimates from 5-minute resting-state recordings for control participants (blue) and patients with chronic pain (red). Frequency ranges were defined as follows: theta (4–7.5 Hz), alpha (8–12.5 Hz), beta (13–30 Hz), low gamma (30.5–60 Hz), and high gamma (60.5–90 Hz). The shaded areas represent the standard error; an independent *t* test (FDR corrected) did not show significant differences between the 2 groups. FDR, false discovery rate.

Visual inspection of the whole-head average sensor PSD for patients with chronic pain showed a lower alpha peak frequency than in control participants (Fig. 1). The PSD for patients with chronic pain showed 2 separate peaks, one at 8.25 Hz and one at 9.25 Hz, whereas the PSD for control participants showed only one peak at 10.25 Hz. Individual sensor-wise averaged PSDs for patients with chronic pain showed that 4 patients contributed to the 8.25 Hz peak, 11 patients contributed to the 9.25 Hz peak, 4 patients contributed to both peaks, whereas 2 patients did not show a clear alpha peak. The average power in the alpha band was lower in patients with chronic pain, however with a large variability across participants. A permutation *t* test (FDR corrected) across all frequency bins did not show significant differences between the sensor-wise averages of the 2 groups (*P* > 0.05).

**Figure 2.**
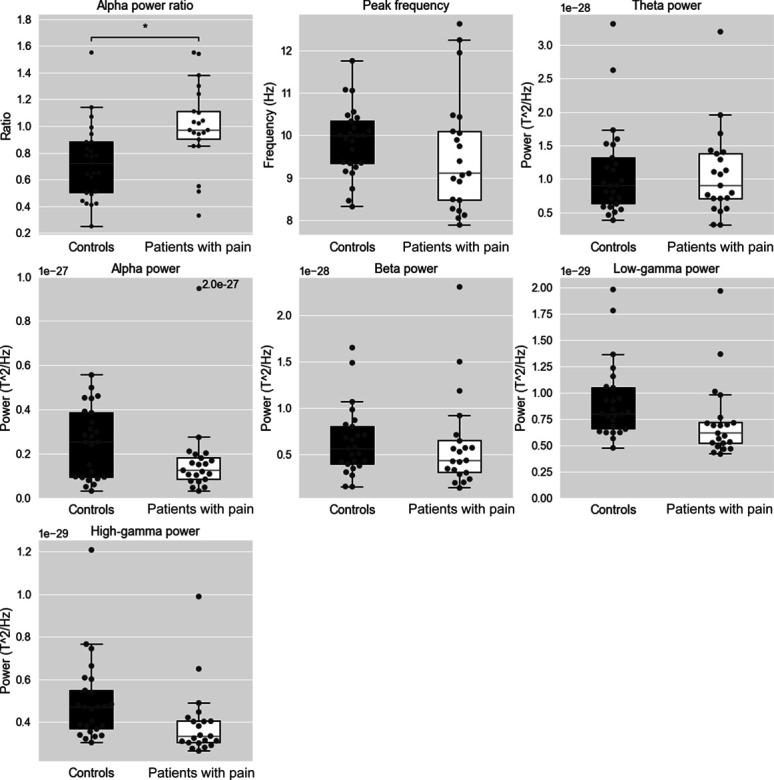
Box plots of the sensor-wise average alpha peak frequency, alpha power ratio, and average power in the theta (4–7.5 Hz), alpha (8–12.5 Hz), beta (13–30 Hz), low-gamma (30.5–60 Hz), and high-gamma (60.5–90 Hz bands for the control participants and the patients with chronic pain. Each measure was averaged over all 275 MEG channels. Whiskers represent 1.5 times the inner quartile range. *P < 0.05, independent *t* test. MEG, magnetoencephalography.

The distribution of group-wise average (across all sensors) alpha peak frequency, alpha power ratio, and power in the theta, alpha, beta, low-gamma, and high-gamma frequency bands are shown in Figure 2. There were no significant differences between the 2 groups in alpha peak frequency or average theta, alpha, beta, low-gamma, or high-gamma power for any sensor. The sensor-wise average of alpha power ratios was significantly higher (*P* = 0.003) in patients with chronic pain (1.00 ± 0.31, mean ± SD) than that in control participants (0.73 ± 0.29).

**Figure 3.**
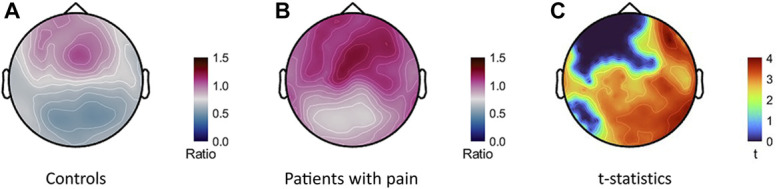
Magnetoencephalography sensor topographies of group average alpha power ratios for (A) control participants and (B) patients with chronic pain, and (C) *t-*statistics (FDR corrected) of the patients with pain vs controls contrast in alpha power ratios. As the statistics did not show negative *t*-values, we plotted positive values only. FDR, false discovery rate.

The sensor topography of alpha power ratios showed stronger values in patients with chronic pain over parietal, occipital, and right lateral sensor regions (Fig. 3, *P* < 0.05, FDR corrected).

Post hoc analyses did not show significant correlations between the alpha power ratio and pain intensity or pain duration measures. The sensor-wise average of alpha power ratios was not significantly correlated with VAS 24 hours before the MEG recording (Pearson correlation coefficient (*r*) = 0.13, *P* = 0.58) or VAS scores collected right before the MEG session (*r* = 0.089, *P* = 0.7). The alpha power ratio did not correlate significantly with pain duration (*r* = 0.29, *P* = 0.21).

### 3.3. Power spectral density analysis: magnetoencephalography source mapping

**Figure 4.**
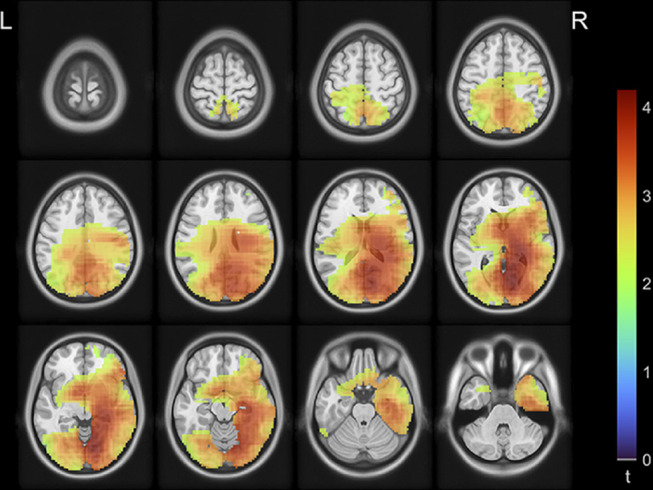
Magnetoencephalography source images for *t*-statistics of patients with chronic pain vs controls contrast in alpha power ratios at each grid point. Only the *t*-values of the grid points that showed a significantly (*P* < 0.05, FDR corrected) higher alpha power ratio for patients with chronic pain were plotted. As the statistics did not show negative *t*-values, we plotted positive values only. FDR, false discovery rate.

None of the 84 AAL brain regions showed statistically significant between-group differences in alpha peak frequency or average power in the tested frequency bands of interest. However, alpha power ratios in multiple brain areas were significantly higher in patients with chronic pain (*P* < 0.05, FDR corrected). These regions were in the occipital, parietal, (mostly right) temporal and (right inferior) frontal, insular and cingulate cortices, and right thalamus (Supplementary Table 1, available at http://links.lww.com/PR9/A107; Fig. 4).

## 4. Discussion

### 4.1. Main findings

Our data show significantly higher alpha power ratios in patients with chronic pain. MEG brain maps showed group-wise significant differences in alpha power ratio over the occipital, parietal, (mostly right) temporal and (right inferior) frontal lobe areas, insular and cingulate cortices, and right thalamus. Overall, these findings indicate regions beyond those typically reported as pain-processing areas as signaling chronic pain phenotypes.

### 4.2. Mapping of alpha power ratios

Several EEG and MEG resting-state studies of chronic pain have previously reported on changes of theta–alpha activity in a diversity of brain regions. Sarnthein et al. pointed at lower dominant frequencies in patients with neuropathic pain with elevated coherence between thalamic local field potentials (LFPs) and scalp EEG.^21,22^ Stern et al. reported overactivation in the high theta (6–9 Hz) frequency range in the bilateral posterior insular, adjacent peri-insular, and inferior posterior parietal cortex.^24^ Slowing of EEG peak frequency was also reported in spinal cord–injured patients with neuropathic pain, primarily in occipital and parietal areas.^5,35^ De Vries et al. reported lower alpha peak frequencies in patients suffering from chronic pancreatitis,^8^ across the scalp, with stronger effects over parietal and occipital regions. Lim et al.^15^ recorded resting-state MEG in patients with fibromyalgia and also reported lower dominant alpha peak frequency localized over the left dorsolateral prefrontal and orbitofrontal cortices. Kim et al.^14^ reported an increased alpha band power and decreased power in the beta band in patients with multiple sclerosis (MS) compared with controls. In their subgroup of patients with MS with neuropathic pain, they found a trend of alpha peak frequency slowing in the thalamus, posterior insula, and posterior cingulate cortex.

Although most of these studies consistently reported a lower dominant (alpha) frequency, shifting towards the theta range, the etiology of pain and the brain regions affected varied substantially between authors. Our data showed a significantly higher alpha power ratio for patients with chronic pain, which is compatible with a lower dominant frequency. We found increased alpha power ratios widespread across the occipital, parietal, (mostly right) temporal and (right inferior) frontal lobe areas, insular and cingulate cortices, and right thalamus. The largest differences were found around subcortical areas, including the thalamus (Supplementary Table 1, available at http://links.lww.com/PR9/A107). Although MEG signals from subcortical structures are weaker, there is growing evidence of methods' advances in detecting activity from deeper brain structures.^6,19^ Previous EEG studies also suggested involvement of the thalamus: Sarnthein et al.^21^ used thalamic LFPs and scalp EEG and showed high coherence between LFP and scalp EEG in the theta range (6–9 Hz), and Stern et al.^24^ showed a normalization of slowed EEG activity after therapeutic thalamic lesions. Our data and these previous studies suggest, in line with the framework of TCD, that relative increases in lower frequency power may have thalamic origins and involve widespread cortical areas.

Strikingly, most brain map differences were localized to the right hemisphere. Although all patients suffered from chronic back pain, the apparent lateralization may be biased by the fact that only a small number of patients (n = 3) suffered from radiating pain exclusively on the right side of their body. Most participants reported radiating pain on both sides (n = 8) or the left side alone (n = 7) and 3 patients reported to have back pain alone. An alternative explanation may point at a greater involvement of right hemisphere structures in chronic pain processes, although the previously mentioned studies of slowing did not report lateralization effects. In acute pain however, neuroimaging effects are mostly contralateral, with the exception of right hemisphere activations in the thalamus, inferior parietal cortex, dorsolateral prefrontal cortex, and dorsal frontal cortex.^7^ Symonds et al. also reported right lateralization of the middle frontal gyrus, anterior cingulate, inferior frontal gyrus, medial or superior frontal gyri, and inferior parietal lobule in an acute pain study.^25^ We tried to quantify lateralization by computing a lateralization index (LI) for the alpha power ratio.^16^ However, LIs were very low (−0.05 < LI < 0.05) and there were no significant differences in LIs between groups. Nevertheless, several areas (eg, thalamus and temporal or frontal areas) showed a significant increase of alpha power ratio in the right hemisphere alone.

To summarize, multiple regions are typically reported to be involved in expressing relatively more signal power in the lower frequencies. These include cingulate, insular, somatosensory, prefrontal cortices, portions of the parietal and occipital cortices, and the thalamus. Our data show expanded contributions from parietal, occipital and temporal lobules, with a trend towards right hemisphere regions. Previous studies have extracted features from resting-state EEG in machine-learning approaches to distinguish between patients with chronic pain and controls.^26,33,34^ Although these studies show promising results, our data indicate that in the search for a chronic pain biomarker we might benefit from including more parietal, occipital, and temporal areas.

The alpha power ratio did not correlate significantly with pain intensity or pain duration. Therefore, if the alpha power ratio measure is indicative of chronic pain, it is not a qualitative marker of perceived pain intensity.

## 5. Limitations

A common bias in pain research stems from depression as a comorbidity of chronic pain. We captured depressive symptoms using the HADS and EQ5D scoring systems, which indeed showed significant differences between patients with chronic pain and controls (Table 1). Newson and Thiagarajan reviewed 18 resting-state EEG studies of depression and reported a general increase in theta-band power in patients.^20^ Increased theta power in comorbid depression could be confounded with increased power in the low alpha band because of spectral proximity. Nonspecific depressive symptoms could therefore cause the alpha power ratio increases in chronic pain and therefore bias the interpretation of our results. We performed post hoc analyses to study the correlation between HADS depression scores and the alpha power ratio. We did not find statistically significant correlations between HADS depression scores and sensor-wise averages of alpha power ratios in patients with chronic pain (r = 0.26, *P* = 0.26). In our control group, we found a statistically significant correlation between global alpha power ratio and HADS depression scores (r = 0.67, *P* < 0.01). However, Vanneste reported that pain and depression should still be distinguishable because they involved distinct sets of brain regions.^33^ Therefore, we correlated HADS depression scores with alpha power ratios of specific brain areas for control participants: The HADS depression score correlated statistically significantly with the alpha power ratio in the posterior cingulate cortex (r = 0.49, *P* = 0.01), right dorsal insula (r = 0.58, *P* < 0.01), and anterior insula (r = 0.60, *P* < 0.01). However, they did not correlate significantly with alpha power ratios in the dorsal anterior cingulate cortex (r = 0.33, *P* = 0.11) and the somatosensory cortex (left: r = 0.27, *P* = 0.20, right: r = 0.14, *P* = 0.51). This suggests that a chronic pain biomarker would benefit from brain region-specific spectral profiles.

Varied analgesic medication usage by patients with chronic pain is also a possible factor of confound. All but 5 of the patients with chronic pain used analgesic medication, but the types and doses of medication varied across participants. This might have affected our results. For example, tramadol has been reported to decrease signal power in the higher range of the alpha band (10.5–12.5 Hz) and alter the expression of lower alpha (8.5–10.5 Hz) and higher beta (18.5–21.0 Hz) signals.^18^

Another limitation might be with the sole inclusion of patients with FBSS chronic pain. This patient group was selected because it is the most common condition seen in our pain clinic. Failed back surgery syndrome is a mixed pain condition consisting of a myriad of surgical and nonsurgical etiologies. Therefore, the patient group with FBSS represents a diverse population with chronic pain. Previous studies showed relative increases in lower-frequency power in neuropathic pain,^22,24^ neuropathic pain due to spinal cord injury,^5,35^ chronic pancreatitis,^8^ fibromyalgia,^15^ and MS.^14^ These observations confirm the hypothesis of slowed activity in patients with chronic pain, although the localization of slowing varied among studies. Further studies are necessary to explore the generalizability of slowing as a marker of chronic pain conditions.

## 6. Conclusion

This exploratory study shows that patients with chronic pain express relatively more signal power in the lower alpha range than matched control participants. This effect was detected using a relative measure: the ratio of power in slow (7–9 Hz) to fast (9–11 Hz) alpha range. Increases in this ratio were not only localized to known pain-processing areas, such as the right thalamus, cingulate, and insular cortex, but also beyond: parietal, occipital, and (mostly right) temporal and (right inferior) frontal lobules. We conclude that the alpha power ratio is a promising signal biomarker pointing at an expanded range of cortical and subcortical regions affected by chronic pain syndromes. Further studies should be conducted to validate these findings.

## Disclosures

The authors have no conflicts of interest to declare.

## Appendix A. Supplemental digital content

Supplemental digital content associated with this article can be found online at http://links.lww.com/PR9/A107.

## Supplementary Material

SUPPLEMENTARY MATERIAL
