# Supplementary material for: Magnetoencephalography reveals increased slow-to-fast alpha power ratios in patients with chronic pain
Source: Pain Rep. 2021 Jun 3;6(2):e928. doi: 10.1097/PR9.0000000000000928 (PMC8177875; doi:10.1097/PR9.0000000000000928)
Supplement: SUPPLEMENTARY MATERIAL [file painreports-6-e928-s001.docx]

Supplementary Table 1: p-value and difference between chronic pain patients (n=21) and controls (n=25) in mean alpha power ratio for each of the 84 brain regions.

| **Brain region** | **p-value** | **Difference in alpha power ratio** |
| --- | --- | --- |
| Posterior cingulate gyrus R | <0.001 | 0.32 |
| Calcarine fissure and surrounding cortex R | <0.001 | 0.33 |
| Inferior temporal gyrus R | <0.001 | 0.38 |
| Cuneus R | 0.001 | 0.30 |
| Hippocampus R | 0.001 | 0.41 |
| Thalamus R | 0.001 | 0.29 |
| Fusiform gyrus R | 0.001 | 0.36 |
| Precuneus R | 0.001 | 0.31 |
| Superior occipital gyrus R | 0.001 | 0.27 |
| Lingual gyrus R | 0.001 | 0.31 |
| Middle temporal gyrus R | 0.001 | 0.34 |
| Caudate nucleus L | 0.001 | 0.27 |
| Posterior cingulate gyrus L | 0.001 | 0.30 |
| Inferior occipital gyrus R | 0.001 | 0.36 |
| Angular gyrus R | 0.001 | 0.34 |
| Middle occipital gyrus R | 0.001 | 0.27 |
| Supramarginal gyrus R | 0.002 | 0.35 |
| Lenticular nucleus, Putamen R | 0.002 | 0.29 |
| Precuneus L | 0.002 | 0.30 |
| Caudate nucleus R | 0.002 | 0.26 |
| Superior temporal gyrus R | 0.002 | 0.36 |
| Middle cingulate & paracingulate gyri R | 0.003 | 0.26 |
| Inferior frontal gyrus, triangular part R | 0.003 | 0.24 |
| Parahippocampal gyrus R | 0.003 | 0.38 |
| Olfactory cortex L | 0.003 | 0.28 |
| Insula R | 0.004 | 0.28 |
| Middle occipital gyrus L | 0.004 | 0.27 |
| Cuneus L | 0.004 | 0.25 |
| Postcentral gyrus L | 0.004 | 0.29 |
| Lenticular nucleus, Putamen L | 0.005 | 0.27 |
| Postcentral gyrus R | 0.005 | 0.29 |
| Calcarine fissure and surrounding cortex L | 0.006 | 0.26 |
| Middle cingulate & paracingulate gyri L | 0.006 | 0.25 |
| Precentral gyrus R | 0.007 | 0.25 |
| Olfactory cortex R | 0.007 | 0.26 |
| Superior parietal gyrus L | 0.008 | 0.30 |
| Paracentral lobule R | 0.008 | 0.24 |
| Superior parietal gyrus R | 0.008 | 0.25 |
| Inferior frontal gyrus, orbital part R | 0.009 | 0.26 |
| Inferior frontal gyrus, opercular part R | 0.009 | 0.25 |
| Inferior occipital gyrus L | 0.009 | 0.28 |
| Inferior parietal gyrus* | 0.010 | 0.24 |
| Lingual gyrus L | 0.011 | 0.24 |
| Insula L | 0.011 | 0.27 |
| Superior occipital gyrus L | 0.012 | 0.23 |
| Paracentral lobule L | 0.015 | 0.22 |
| Precentral gyrus L | 0.019 | 0.24 |
| Temporal pole: superior temporal gyrus R | 0.020 | 0.25 |
| Rolandic operculum R | 0.020 | 0.30 |
| Temporal pole: middle temporal gyrus R | 0.021 | 0.28 |
| Rolandic operculum L | 0.021 | 0.22 |
| Inferior parietal gyrus* | 0.022 | 0.26 |
| Angular gyrus L | 0.024 | 0.27 |
| Anterior cingulate & paracingulate gyri L | 0.025 | 0.18 |
| Anterior cingulate & paracingulate gyri R | >0.050 | 0.15 |
| Inferior frontal gyrus, opercular part L | >0.050 | 0.22 |
| Inferior frontal gyrus, orbital part L | >0.050 | 0.15 |
| Inferior frontal gyrus, triangular part L | >0.050 | 0.18 |
| Superior frontal gyrus, medial orbital L | >0.050 | 0.12 |
| Superior frontal gyrus, medial orbital R | >0.050 | 0.15 |
| Middle frontal gyrus L | >0.050 | 0.18 |
| Middle frontal gyrus R | >0.050 | 0.17 |
| Middle frontal gyrus, orbital part L | >0.050 | 0.15 |
| Middle frontal gyrus, orbital part R | >0.050 | 0.21 |
| Superior frontal gyrus, dorsolateral L | >0.050 | 0.16 |
| Superior frontal gyrus, dorsolateral R | >0.050 | 0.16 |
| Superior frontal gyrus, medial L | >0.050 | 0.09 |
| Superior frontal gyrus, medial R | >0.050 | 0.06 |
| Superior frontal gyrus, orbital part L | >0.050 | 0.14 |
| Superior frontal gyrus, orbital part R | >0.050 | 0.16 |
| Fusiform gyrus L | >0.050 | 0.13 |
| Hippocampus L | >0.050 | 0.17 |
| Parahippocampal gyrus L | >0.050 | 0.17 |
| Gyrus rectus L | >0.050 | 0.19 |
| Gyrus rectus R | >0.050 | 0.21 |
| Supplementary motor area L | >0.050 | 0.21 |
| Supplementary motor area R | >0.050 | 0.21 |
| Supramarginal gyrus L | >0.050 | 0.29 |
| Inferior temporal gyrus L | >0.050 | 0.20 |
| Middle temporal gyrus L | >0.050 | 0.21 |
| Temporal pole: middle temporal gyrus L | >0.050 | 0.26 |
| Temporal pole: superior temporal gyrus L | >0.050 | 0.27 |
| Superior temporal gyrus L | >0.050 | 0.15 |
| Thalamus L | >0.050 | 0.17 |

*excluding supramarginal and angular gyri
